# High-Frequency Ipsilesional versus Low-Frequency Contralesional Transcranial Magnetic Stimulation after Stroke: Differential Effects on Ipsilesional Upper Extremity Motor Recovery

**DOI:** 10.3390/medicina59111955

**Published:** 2023-11-06

**Authors:** Laura Petruseviciene, Alexander T. Sack, Raimondas Kubilius, Raimondas Savickas

**Affiliations:** 1 Department of Rehabilitation, Lithuanian University of Health Sciences, 44307 Kaunas, Lithuania; raimondas.kubilius@lsmuni.lt (R.K.); raimondas.savickas@lsmuni.lt (R.S.); 2 Department of Physical Medicine and Rehabilitation, Hospital of Lithuanian University of Health Sciences Kaunas Clinics, 50161 Kaunas, Lithuania; 3 Faculty of Psychology and Neuroscience, Maastricht University, 6229 ER Maastricht, The Netherlands; a.sack@maastrichtuniversity.nl

**Keywords:** transcranial magnetic stimulation, stroke rehabilitation, affected upper extremity, unaffected upper extremity, neurorehabilitation

## Abstract

*Background and Objectives*: Stroke is a major cause of death and disability worldwide; therefore, transcranial magnetic stimulation (TMS) is being widely studied and clinically applied to improve motor deficits in the affected arm. However, recent studies indicate that the function of both arms can be affected after stroke. It currently remains unknown how various TMS methods affect the function of the ipsilesional upper extremity. *Materials and Methods*: Thirty-five subacute stroke patients with upper extremity motor deficits were enrolled in this study and randomly allocated into three groups, receiving either (1) low-frequency rTMS over the contralesional hemisphere; (2) high-frequency rTMS over the ipsilesional hemisphere; or (3) no stimulation. Experimental groups received 10 rTMS sessions over two weeks alongside standard rehabilitation, and the control group received the same procedures except for rTMS. Both affected and unaffected upper extremity motor function was evaluated using hand grip strength and Functional Independence Measure (FIM) tests before and after rehabilitation (7 weeks apart). *Results*: All groups showed significant improvement in both the affected and unaffected hand grip and FIM scores (*p* < 0.05). HF-rTMS led to a notably higher increase in unaffected hand grip strength than the control group (*p* = 0.007). There was no difference in the improvement in affected upper extremity motor function between the groups. The FIM score increase was lower in the control group compared to experimental groups, although not statistically significant. *Conclusions*: This study demonstrates the positive effect of ipsilesional HF-rTMS on the improvement in unaffected arm motor function and reveals the positive effect of both LF- and HF-rTMS on the affected upper extremity motor function recovery.

## 1. Introduction

Stroke remains the second leading cause of death [1] and one of the leading causes of severe long-term adult disability worldwide [2,3]. The incidence rate of stroke increased almost twice during the past three decades, affecting 12.2 million people annually worldwide [1]. Moreover, due to an increase in conventional stroke risk factors across the entire age range, such as hypertension, hyperlipidemia, smoking, and obesity, there has been an increase in stroke incidence in the young [3]. Approximately 80% of stroke survivors suffer from impaired upper extremity motor function [4,5]. Only 30% to 50% of these stroke survivors regain functional hand movements within six months after stroke [5,6] and the majority are left with disabling neurological deficits [7]. This emphasized the need for new, improved treatment options to better support the rehabilitation of these patients. To address this need, scientists have increasingly focused on non-invasive brain stimulation technologies capable of modulating brain excitability in order to improve motor deficits in subacute and chronic stroke patients. One such method is repetitive transcranial magnetic stimulation (rTMS), an established brain technique using electromagnetic stimulation to assess and alter cortical excitability and network connectivity in the healthy and diseased brain [5,6,8].

In a healthy brain, unilateral activation of the primary motor cortex (M1) induces inhibitory effects on contralateral M1 through transcallosal pathways [9]. The balance of this interhemispheric inhibition (IHI) has been shown to be impaired after unilateral stroke [10]. As a consequence of damaged intracortical connections, contralesional M1 can be disinhibited due to reduced inter-hemispheric suppression from the damaged ipsilesional M1, resulting in pathological overexcitability of the unaffected motor cortex. This disinhibition of the unaffected hemisphere may directly affect motor function recovery [9,11,12]. Depending on the frequency with which rTMS is applied, cortical excitability can be either suppressed or facilitated [13]. Low frequency (≤1 Hz, low-frequency (LF)-rTMS) has been shown to reduce and high frequency (>1 Hz, high-frequency (HF)-rTMS) has been shown to increase the excitability of the targeted brain region [13,14]. Following this rationale, it has previously been shown that both LF-rTMS applied to the contralesional hemisphere as well as HF-rTMS applied to the ipsilesional hemisphere can have a positive effect on the affected contralesional upper extremity motor function recovery after ischemic stroke. It, however, remains largely unclear which of these two different protocols (LF versus HF) applied to which hemisphere (ipsilesional or contralesional) is the most effective [13,15,16,17,18]. Interestingly and also following the logic of inter-hemispheric suppression, effects of either rTMS protocol should not exclusively be monitored for the affected limb contralateral to the site of the lesion, but should also include assessing possible effects on the ipsilesional arm function.

An increasing number of studies indicate that the function of the ipsilesional arm is also affected after unilateral stroke likely because 10% to 15% of corticospinal pathways run uncrossed through the spinal cord to the end muscles [19,20]. Yet, in stroke rehabilitation, little focus has been targeted toward the ipsilesional arm function and the impact of its deficit on functional independence [19,21], nor did the previous rTMS studies systematically report effects on both affected and unaffected arm function.

This present study aimed to evaluate the relative effect of different rTMS treatment approaches (LF versus HF) on both ipsilesional and contralesional extremity motor function recovery in subacute stroke patients in the context of a treatment such as a usual rehabilitation program.

## 2. Materials and Methods

### 2.1. Participants

Thirty-five subacute stroke patients with upper extremity motor deficits were enrolled in this study according to the following criteria: (1) ischemic stroke of the middle cerebral artery (MCA) or ischemic vertebrobasilar (VB) stroke, confirmed through instrumental tests (CT, MRI); (2) acute hemiplegia/hemiparesis, hand motor deficit, muscle strength ≤ 4 points (as assessed by the Lovett scale); (3) time after the stroke before inclusion in this study is no more than 1 month; (4) no severe deficit in cognitive functional (a Mini-Mental State Examination (MMSE) ≥ 18 points); (5) no contraindications of rTMS; and (6) 18 years and older. Exclusion criteria were as follows: (1) patients with implanted ferromagnetic or other metal devices sensitive to a magnetic field in the head or neck area; cochlear implants; implanted neurostimulators, pacemakers, or drug delivery pumps; (2) complete aphasia or severe cognitive impairment (a Mini-Mental State Examination (MMSE) < 18 points); (3) taking tricyclic antidepressants, neuroleptics, or benzodiazepines; (4) previous skull fractures or other head injuries with loss of consciousness; (5) history of epilepsy or seizures; (6) spasticity of the upper limb (Ashworth scale > 2); and (7) pregnancy. This study was conducted in accordance with the Declaration of Helsinki [22].

This study was approved by Kaunas Regional Biomedical Research Ethics Committee (No: BE-2-86). All participants gave informed consent before the experiment.

In this study, we present findings derived from an ongoing clinical trial registered on clinicaltrials.gov, ID: NCT05646134.

### 2.2. Study Design

Based on a randomized, single-blind controlled trial, all patients who were admitted to the Neurorehabilitation department between December 2021 and December 2022 and met the inclusion criteria were enrolled in this study and were randomly allocated into three groups, receiving either (1) low-frequency rTMS over contralesional hemisphere; (2) high-frequency rTMS over ipsilesional hemisphere; or (3) no stimulation. As none of the patients had undergone TMS treatment before, individuals in the experimental groups were blinded to their group allocation. All participants in the experimental groups received 10 sessions of rTMS over two weeks along with routine rehabilitation procedures: physiotherapy and occupational therapy for both affected and less affected arms, massage of the affected extremities, electrostimulation, and psychological consultations. Participants in the control group received the same procedures except for rTMS treatment. Both affected and unaffected upper extremity motor function was evaluated through the hand grip strength [23] test performed using a digital hand-held dynamometer at the beginning and the end of rehabilitation (7 weeks apart). In addition, the Functional Independence Measure (FIM) test [24] was performed before and after the rehabilitation to evaluate the functional independence of participants.

### 2.3. Intervention

Repetitive transcranial magnetic stimulation procedures were performed using a Magstim^®^ Rapid2 stimulator (Software number: 3473, Version: V13.0), equipped with an eight-figure coil. At the beginning of the procedure, the primary motor cortex (M1) and resting motor threshold (RMT) were established. A resting motor threshold (RMT) was defined as the lowest intensity that could elicit any time-locked movement caudal to the wrist in five out of ten trials [14]. In the LF-RTMS group, stimulation was performed at 1 Hz, 80% of RMT, over the M1 of the contralesional hemisphere, applying a total of 1200 pulses (10 trains, 120 pulses per train, intertrain interval 20 s). In the HF-RTMS group, stimulation was performed at 10 Hz, 80% of RMT, over the M1 of the ipsilesional hemisphere in a total of 1200 pulses (30 trains, 40 pulses per train, intertrain interval 20 s). Subjects in the control group did not receive rTMS intervention. All participants received the same routine rehabilitation procedures: physiotherapy, occupational therapy, massage of the affected extremities, electrostimulation, and psychological consultations.

### 2.4. Statistical Analysis

Statistical analysis of the data was performed using the statistical software package IBM SPSS 22.0. When analyzing the data, descriptive numerical characteristics were calculated: the number of cases, median, and 25th–75th percentiles (Q1–Q3). Qualitative non-parametric criteria were assessed using the chi-square (χ^2^) test, and quantitative non-parametric criteria were assessed using the Kruskal–Wallis test. The significance was set at *p* < 0.05.

## 3. Results

**Figure 1:**
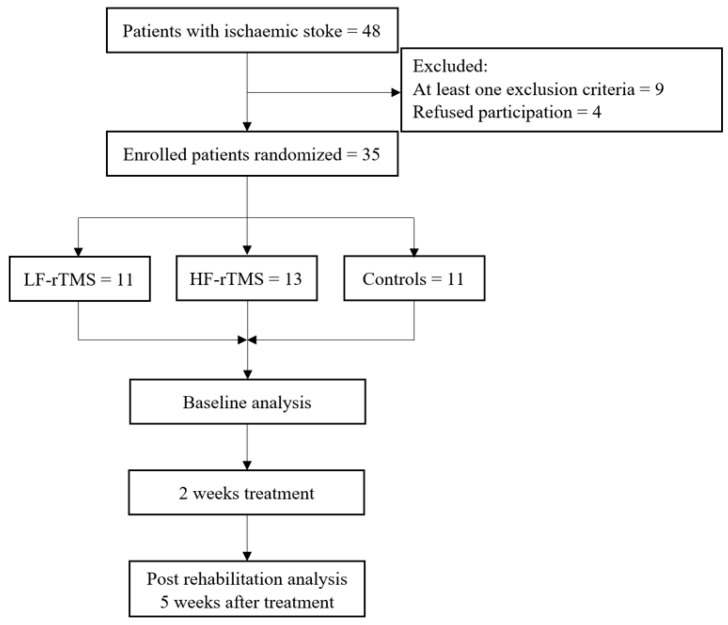
Study flow chart.

Forty-eight patients with a history of first-ever unilateral ischemic stroke, admitted at the Neurorehabilitation department of the Hospital of Lithuanian University of Health Sciences Kaunas Clinics for standard stroke rehabilitation from December 2021 to December 2022, were screened for inclusion in this study. Thirty-five patients that met the criteria and gave written informed consent were enrolled in this study and randomly allocated into three groups: (1) contralesional LF-rTMS (*n* = 11), (2) ipsilesional HF-rTMS (*n* = 13), and a control group (*n* = 11). A flow chart showing inclusion into this study is presented in Figure 1.

Demographic characteristics and baseline values between the groups are summarized in Table 1. There were no significant differences among the groups.

There were no differences among groups before treatment with regard to unaffected upper extremity hand grip strength (*p* = 0.11), affected upper extremity hand grip strength (*p* = 0.12), and FIM score (*p* = 0.15). After the treatment, both affected and unaffected upper extremity hand grip and FIM scores were significantly improved in all groups (*p* < 0.05). The exact numbers and *p* values are presented in Table 2.

Unaffected upper extremity hand grip strength significantly increased more in the HF-rTMS group compared to the control group (*p* = 0.007). There was no difference in the improvement in affected upper extremity motor function between the groups. The FIM score increased the least in the control group compared to both LF- and HF-rTMS groups, although the differences were not statistically significant. The exact numbers and *p* values are presented in Table 3.

## 4. Discussion

In this study, we aimed to evaluate which of the currently applied brain stimulation approaches for motor stroke rehabilitation is more effective, using high-frequency rTMS to increase the excitability of the affected ipsilesional hemisphere, or using low-frequency rTMS to reduce the disinhibited hyperactivity within the unaffected contralesional hemisphere. To this end, we directly compared those two approaches with a control group not receiving brain stimulation in the rehabilitation program for subacute stroke. Also, we systematically assessed effects of rTMS on both the ipsilesional and contralesional upper extremities motor function.

A significantly improved motor function of both affected and unaffected upper extremities and functional independence were observed in all groups after 7 weeks of rehabilitation. However, opposite from what was expected, none of the two rTMS approaches significantly added an additional improvement in upper extremity motor function or functional independence when applied as an add-on therapy to the standard rehabilitation program (control group). Yet, we analyzed the rTMS effect on top of an already effective stroke rehabilitation program; hence, it is more difficult to obtain significant findings. Importantly, we found that HF-rTMS (10 Hz) applied to the ipsilesional hemisphere significantly improved the unaffected upper extremity hand grip strength compared to the control group. This is intriguing and potentially relevant as we were unable to identify other studies that found motor function improvements of the unaffected upper extremity after a course of rTMS treatment, as we report here. Most previous rTMS studies either evaluated the motor function of only the affected arm [15,16,25,26,27,28] or did not observe any significant differences in unaffected upper extremity motor recovery [16,29]. Despite the fact that rTMS has been poorly investigated on ipsilesional arm motor recovery after stroke, our findings suggest that it might be an effective treatment of both affected and unaffected upper extremities. This nicely aligns with the increasing number of studies indicating ipsilesional upper extremity impairments after stroke [19], and the recent suggestion that the grip strength of the unaffected arm might be a predictor for short-term motor recovery after stroke with motor training of the unaffected arm having a significant correlation with the functional outcome [30].

After unilateral stroke, the affected side of the brain can cause dysfunction in both contralesional and ipsilesional arms. It is believed to occur because approximately 10% to 15% of corticospinal pathways travel uncrossed directly from the brain to the end muscles of the body. The other reason is a phenomenon known as diaschisis, in which a weakened connection between two areas of the brain caused by damage to one area can lead to reduced function in the other area [19]. While not as obvious as contralesional deficits, ipsilesional deficits can have a major impact on post-stroke functional recovery since during many activities of daily living, both limbs are required, and the presence of ipsilesional impairments can further hinder an individual’s ability to complete these tasks [19,31]. Additionally, it is acknowledged that ipsilesional arm dysfunction is closely related to the severity of the stroke, as the higher the damage to the brain, the more likely decreased ipsilesional function is caused [21], as well as with lateralization of the stroke, as a subject with a stroke in the right hemisphere more often suffers from reduced motor function of both contra- and ipsilesional arms [21,32]. However, impaired function of the ipsilesional arm after a unilateral stroke is often overlooked and consequently left untreated [31,33,34].

Our study shows that ipsilesional HF-rTMS has a significantly better effect on unaffected upper extremity motor recovery than both LF-rTMS and sham stimulation. We believe that the primary cause of this outcome is likely diaschisis, which occurs because of damaged nerve fibers. This damage leads to a reduction in secondary blood flow in the hemisphere on the same side as the injury [35]. Regarding this phenomenon, excitatory stimulation might increase blood flow, oxygen metabolic rate, cerebral glucose metabolic rate, and other parameters in both hemispheres via neural connections, resulting in an improved function of both upper extremities. Naturally, this raises the question if excitatory rTMS over the intact cortex had even better results for motor recovery. We managed to find only one study addressing this hypothesis [36]. The results of that study suggest that the modulation of abnormal interhemispheric inhibition might be useful for patients with mild motor dysfunction but may be less effective for those with severe deficits due to extensive damage of transcallosal pathways. Therefore, the treatment that can stimulate the compensatory effects might be superior to the treatment that modulates the excitability of the brain cortex in severe stroke. However, if HF-rTMS has excitatory effects on both hemispheres, does LF-rTMS have the opposite suppressor effect? Because LF decreases the excitability of the intact cortex, some researchers anticipated that it could produce a reverse effect on the unaffected upper extremity; however, a meta-analysis performed in 2014 ruled out this possible adverse effect, and to our knowledge, this question was not discussed in future studies [37]. We also believe that LF-rTMS is safe for both affected and unaffected upper extremity motor recovery. However, based on our current findings, it might not have an impact on compensatory effects in the intact cortex and might thus not improve ipsilesional arm function.

Similar to the results of other authors [38,39], in our study, the tendency of both LF- and HF-rTMS treatment to increase the functional independence of patients was observed, even though, in our case, there was no significant difference compared to the control group.

Our study was not free of limitations. We believe the absence of significantly better results of both hand grip strength and Functional Independence Measure tests in experimental groups was due to the small sample. Despite the small sample, we also performed a small number of tests for upper extremity motor function evaluation. Even though grip strength is considered to be a good indicator of hand function after stroke [30], more tests should be performed since upper extremity function depends not only on muscle strength but also on dexterity, spasticity, range of motion, and proprioception. Many scientists perform a Fugl Meyer Assessment for Upper Extremity (FMA-UE) and Wolf Motor Function Test (WMFT) in stroke rehabilitation since both of these tests are stroke-specific. Still, FMA-UE is believed to be more sensitive to changes during the rehabilitation process [40]. A variety of other measures, like the Nine-Hole Peg Test, Box and Block test, Action Research Arm Test, finger tapping, pinch strength, modified Rankin scale, and Barthel index, are being used for evaluation of upper extremity motor function rehabilitation [25,26,27,28,29]. Every test has its own benefits; therefore, using more outcome measures might be helpful for a more accurate assessment of stroke rehabilitation. Moreover, rTMS is known to have a great placebo effect for stroke patients [41], and we did not have an opportunity to control this effect, since we did not apply sham stimulation for the control group. Furthermore, in this study, we did not assess the impact of education, manual dominance, comorbidities, and other baselines values that could have an impact on stroke rehabilitation effectiveness. Assessing baseline values reduces variability, enables comparative analyses, monitors individual responses, and upholds scientific rigor, all of which contribute to a comprehensive and robust assessment of the intervention’s impact [42].

It is crucial to emphasize that although this study presents statistical trends and significance, understanding the clinical and practical implications of these findings necessitates additional investigation. Further research is needed to assess this aspect.

## 5. Conclusions

In conclusion, this study demonstrated the possible effect of both LF- and HF-rTMS on affected upper extremity motor function recovery in stroke patients. However, due to the small sample and limited outcome measures, the results were insignificant. It also showed the differential positive effect of HF-rTMS on the improvement in unaffected arm motor function. This finding requires a better understanding of how rTMS may affect the ipsilesional arm motor function after stroke, an area largely under-investigated in the literature today. Further studies with larger, randomized, controlled samples are needed for a better assessment of the efficacy and safety of rTMS for the recovery of both affected and unaffected upper extremity motor function after a stroke.

## Figures and Tables

**Table 1:** Demographic characteristics of subjects among the groups.

Variables	LF-rTMS (*n* = 11)	HF-rTMS (*n* = 13)	Control (*n* = 11)	*p* Value
Age, years, median (Q1–Q3)	64.00 (54.00–76.00)	66.00 (60.00–71.00)	76.00 (71.00–82.00)	0.07
Gender, Male/Female	7/4	9/4	5/6	0.47
Stroke location, MCA/vertebrobasilar	10/1	11/2	9/2	0.82
Affected side, Right/left	7/4	5/8	7/4	0.35
RMT, median (Q1–Q3)	55.00 (50.00–60.00)	51.00 (39.00–59.00)	-	0.39
Unaffected upper extremity hand grip strength, kg, median (Q1–Q3)	32.00 (29.00–40.00)	36.00 (23.00–44.00)	22.00 (17.00–36.00)	0.11
Affected upper extremity hand grip strength, kg, median (Q1–Q3)	0.00 (0.00–2.00)	3.00 (0.00–12.00)	4.00 (0.00–18.00)	0.21
FIM, score, median (Q1–Q3)	38.00 (34.00–47.00)	44.00 (35.00–55.00)	32.00 (22.00–47.00)	0.15

**Table 2:** Motor tests and functional independence test variables of subjects among the groups before and after the rehabilitation.

Test	Group	Before	After	*p* Value
Unaffected upper extremity hand grip strength, kg, median (Q1–Q3)	LF-rTMS (*n* = 11)	32.00 (29.00–40.00)	41.00 (30.00–48.00)	0.003
	HF-rTMS (*n* = 13)	36.00 (23.00–44.00)	49.00 (30.00–54.00)	0.001
	Control (*n* = 11)	22.00 (17.00–36.00)	24.00 (19.00–38.00)	0.010
Affected upper extremity hand grip strength, kg, median (Q1–Q3)	LF-rTMS (*n* = 11)	0.00 (0.00–2.00)	9.00 (0.00–19.00)	0.018
	HF-rTMS (*n* = 13)	3.00 (0.00–12.00)	9.00 (3.00–21.00)	0.005
	Control (*n* = 11)	4.00 (0.00–18.00)	15.00 (3.00–30.00)	0.011
FIM, score, median (Q1–Q3)	LF-rTMS (*n* = 11)	38.00 (34.00–47.00)	78.00 (59.00–88.00)	0.003
	HF-rTMS (*n* = 13)	44.00 (35.00–55.00)	80.00 (72.00–101.00)	0.001
	Control (*n* = 11)	32.00 (22.00–47.00)	62.00 (45.00–72.00)	0.003

**Table 3:** Motor tests and functional independence test changes among the groups before and after the rehabilitation.

Variables	LF-rTMS (*n* = 11)	HF-rTMS (*n* = 13)	Control (*n* = 11)	*p* Value
Unaffected upper extremity hand grip strength, kg, median (Q1–Q3)	4.00 (2.00–11.00)	8.00 (5.00–12.00) *	2.00 (0.00–2.00) *	0.007
Affected upper extremity hand grip strength, kg, median (Q1–Q3)	9.00 (0.00–10.00)	4.00 (1.00–7.00)	4.00 (0.00–12.00)	0.95
FIM, score, median (Q1–Q3)	35.00 (22.00–49.00)	38.00 (29.00–48.00)	25.00 (15.00–40.00)	0.11

*—statistical significance.

## Data Availability

The data presented in this study are available on request from the corresponding author. The data are not publicly available due to privacy reasons.
